# Expression Analysis of All Protease Genes Reveals Cathepsin K to Be Overexpressed in Glioblastoma

**DOI:** 10.1371/journal.pone.0111819

**Published:** 2014-10-30

**Authors:** Urška Verbovšek, Helena Motaln, Ana Rotter, Nadia A. Atai, Kristina Gruden, Cornelis J. F. Van Noorden, Tamara T. Lah

**Affiliations:** 1 Department of Genetic Toxicology and Cancer Biology, National Institute of Biology, Ljubljana, Slovenia; 2 Department of Biotechnology and Systems Biology, National Institute of Biology, Ljubljana, Slovenia; 3 Department of Cell Biology and Histology, Academic Medical Center, University of Amsterdam, Amsterdam, The Netherlands; 4 Faculty of Chemistry and Chemical Technology, University of Ljubljana, Ljubljana, Slovenia; University of Florida, United States of America

## Abstract

**Background:**

Cancer genome and transcriptome analyses advanced our understanding of cancer biology. We performed transcriptome analysis of all known genes of peptidases also called proteases and their endogenous inhibitors in glioblastoma multiforme (GBM), which is one of the most aggressive and deadly types of brain cancers, where unbalanced proteolysis is associated with tumor progression.

**Methods:**

Comparisons were performed between the transcriptomics of primary GBM tumors and unmatched non-malignant brain tissue, and between GBM cell lines (U87-MG and U373) and a control human astrocyte cell line (NHA). Publicly-available data sets and our own datasets were integrated and normalized using bioinformatics tools to reveal protease and protease inhibitor genes with deregulated expression in both malignant versus non-malignant tissues and cells.

**Results:**

Of the 311 protease genes identified to be differentially expressed in both GBM tissues and cells, 5 genes were highly overexpressed, 2 genes coding for non-peptidase homologues transferrin receptor (*TFRC*) and G protein-coupled receptor 56 (*GPR56*), as well as 3 genes coding for the proteases endoplasmic reticulum aminopeptidase 2 (*ERAP2*), glutamine-fructose-6-phosphate transaminase 2 (*GFPT2*) and cathepsin K (*CTSK*), whereas one gene, that of the serine protease carboxypeptidase E (*CPE*) was strongly reduced in expression. Seventy five protease inhibitor genes were differentially expressed, of which 3 genes were highly overexpressed, the genes coding for stefin B (*CSTB*), peptidase inhibitor 3 (*PI3* also named elafin) and *CD74*. Seven out of 8 genes (except *CSTB*) were validated using RT-qPCR in GBM cell lines. *CTSK* overexpression was validated using RT-qPCR in GBM tissues as well. Cathepsin K immunohistochemical staining and western blotting showed that only proteolytically inactive proforms of cathepsin K were overexpressed in GBM tissues and cells.

**Conclusions:**

The presence of high levels of inactive proforms of cathepsin K in GBM tissues and cells indicate that in GBM the proteolytic/collagenolytic role is not its primary function but it plays rather a different yet unknown role.

## Introduction

Glioblastoma multiforme (GBM) is the most malignant form of glioma with the median survival time of patients being only 15 months after diagnosis [1]. One of the major reasons for the poor prognosis is diffuse infiltration of highly-invasive individual cancer cells into the brain parenchyma that makes complete tumor resection impossible [2]. Proteolytic enzymes (peptidases also called proteases) are associated with invasive growth of cancer including GBM [3]–[6]. Invasion of glioma cells into brain parenchyma is biologically distinct from that in other tissues, because brain extracellular matrix (ECM) differs from ECM of most organs. Due to the compact cellular assembly, it is condensed to approximately 20% of the tissue volume. Brain ECM consists primarily of glycosaminoglycans (GAGs) and proteoglycans mostly without collagen, except in two areas: around blood vessels and at the pial surface (glia limitans) where a basal lamina is present. ECM structure of the brain is reviewed in Rauch (2007) [7].

Proteases may also be involved in processes other than proteolytic activity during invasion that are associated with tumor progression [8], [9], such as proliferation, survival, angiogenesis, senescence, apoptosis and autophagy. Moreover, exosites of proteases can bind to physiologically-relevant partners in proteolytic signaling [10]. These and possibly novel functions can be revealed using bioinformatics tools, providing data integration approaches via identification of deregulated expression of protease and protease inhibitor genes, not previously associated with GBM.

Activity of proteases is tightly regulated at all expression levels, including the post-translational level where endogenous inhibitors and possible interactions with other macromolecules regulate their activity [11]. Besides, the balance between proteases and inhibitors plays a crucial role in the degradation of physiological substrates, altogether forming a molecular web, the so-called cancer degradome [12]. A number of proteases and endogenous protease inhibitors have been implicated in tumor initiation and progression, and either promote or inhibit tumor progression [9].

In GBM, proteases and their inhibitors are involved in regulation of activity of growth factors and chemotactic factors as well as modulation of the cytoskeleton and cellular responses besides their activity in the ECM [10], [11]. Proteases and their inhibitors have been suggested to be biomarkers as well as prognostic and predictive markers for GBM patients [12].

In humans, there are, according to the MEROPS database [13], 1133 known and putative proteases and 1615 known and putative protease inhibitors. This database contains not only genes coding for proteins with confirmed protease activity, but also includes genes that have only domains homologous to known proteolytic domains, the so-called non-peptidase homologues. The aim of the study was to reveal known and putative MEROPS proteases and protease inhibitors that show deregulated expression in GBM. Therefore, GBM and control tissues and cell lines were used to identify proteases of the cancer cells only, excluding normal cells that are present in the tumor microenvironment [9].

The aim of the study was addressed as follow: First, expression levels of genes of proteases and protease inhibitors in human GBM tissues and cell lines versus their non-malignant counterparts were determined using a bioinformatics approach to assess what proteases and protease inhibitors show specifically deregulated expression in GBM cancer cells. Second, because identification of overexpressed genes is more reliable and more valuable for clinical applications such as diagnosis, prognosis and therapy, we only performed validation of the bioinformatics data of selected proteases that were highly overexpressed in GBM tissues and cells. We have performed the expression analysis in GBM tissues *in silico* using publicly-available datasets as we have done previously for all known kinase genes [14], osteopontin [15] and NADP^+^-dependent dehydrogenases [16].

We show that the *CTSK* gene coding for the lysosomal cysteine protease cathepsin K (CatK) is highly overexpressed in both GBM tissues and GBM cells, and this finding was validated by showing high levels of mRNA and protein in these tissues and cells.

## Materials and Methods

### Transcriptomics analyses

#### Cell lines

Human GBM cell lines (U87-MG, U373) were purchased from the American Type Culture Collection (ATCC; Manassas, VA, USA) and cultured according to the recommendations of the supplier. Authentication was performed with DNA fingerprinting, using Amp-FlSTR Profiler Plus PCR Amplification Kit (Applied Biosystems, Life Technologies, Paisley, UK) [17]. Cell lines of passage 40 were used in the experiments.

#### Gene expression microarrays

Total RNA was isolated, purified and quantified from 6 biological repetitions of U87-MG cells and 3 biological repetitions of U373 cells [18]. Total RNA samples were labelled with an Illumina TotalPrep RNA Amplification Kit (Ambion, Life Technologies) and subsequently hybridized to Illumina HumanWG-6 v3 Expression BeadChip (Illumina BeadChip; Illumina, San Diego, CA, USA). After scanning, image acquisition was carried out by applying BeadStudio version 3.3.7 software (Illumina). Data is deposited in NCBI's Gene Expression Omnibus (GEO, http://www.ncbi.nlm.nih.gov/geo/) – series GSE26283 (samples GSM645515, GSM645519 and GSM645523) for U87-MG cells and series GSE59634 (samples GSM1440969, GSM1440973 and GSM1440977) for U373 cells.

#### Gene expression data from databases

Gene expression data of the normal human astrocyte (NHA) cell line, human brain tumor cell lines (U87-MG, U373), GBM tissue and control non-malignant brain tissue was obtained from publicly available GEO, EMBL-EBI's ArrayExpress (http://www.ebi.ac.uk/arrayexpress/) and from The Cancer Genome Atlas (TCGA) Data Portal (https://tcga-data.nci.nih.gov/tcga/). Downloaded gene expression data was obtained using either Affymetrix Human Genome U133 Plus 2.0 Array (Affymetrix GeneChip; Affymetrix, Santa Clara, CA, USA) or Illumina HumanWG-6 v3 Expression BeadChip, Illumina). We used raw data of samples GSM309429, GSM309431 and GSM309432 from GEO series GSE12305 (NHA); samples GSM247617, GSM247618 and GSM247619 from GEO series GSE9834 (NHA); samples GSM460681, GSM460682 and GSM460683 from GEO series GSE18494 (U87-MG); samples U373D-1, U373D-2 and U373D-4 from ArrayExpress series E-MEXP-903 (U373); and GBM tissue (24 samples) and unmatched control brain tissue (10 samples) obtained from the TCGA Data Portal.

#### Data analysis (cross-platform comparison)

Datasets were first pre-processed using variance stabilization normalization [19]. To integrate data from different microarray platforms (Illumina and Affymetrix), normalized data obtained from Affymetrix GeneChips (from public repositories) and from Illumina BeadChips (from our laboratory) were first converted to Entrez gene identifiers (IDs) using DAVID (The Database for Annotation, Visualization and Integrated Discovery; http://david.abcc.ncifcrf.gov/) online tool.

The same Entrez IDs within a microarray were averaged thus giving a single gene expression value for a replicate.

**Figure 1:**
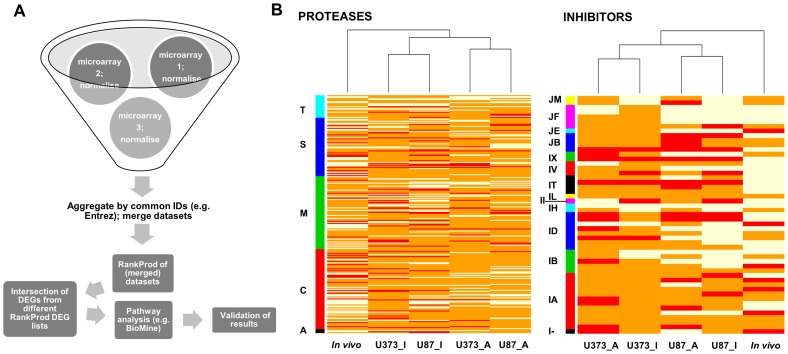
Analysis pipeline (A) and heatmap of differentially-expressed protease and protease inhibitor genes in GBM tissues *(in vivo)* and U373 and U87 cell lines (B). (A) Data from different sources and platforms were integrated after quality control and normalization that were performed separately for each microarray dataset and were converted to a defined identifier (Entrez gene ID). Probes with the same Entrez gene ID were aggregated and averaged and datasets merged with keeping only data when an identifier was found across all microarrays. Differentially-expressed genes were identified by the nonparametric RankProd method applied to merged data and searched for the intersection with a list of proteases and protease inhibitors genes. Hierarchical clustering was done in R statistical software using default parameters (complete linkage method). Afterwards, pathway analysis was performed to assist the biological interpretation of results and the selected candidate genes were validated by molecular biology tools. (B) Heatmap showing differential expression of protease and protease inhibitor genes in GBM tissues as compared to non-malignant brain tissue (*in vivo*) versus two GBM-derived cell lines as compared to NHA cell line. Legend: U87_I, U373_I - data for U87-MG and U373 cell line obtained from Illumina microarray; U87_A, U373_A - data for U87-MG and U373 cell line obtained from Affymetriy microarray; T - threonine proteases, S - serine proteases, M - metalloproteases, C - cysteine proteases, A - aspartic proteases; JM, JF, JE, JB, IX, IV, IT, IL, II, IH, ID, IB, IA, I- - clans of protease inhibitors.

Only data with Entrez IDs appearing in both data sets (Affymetrix and Illumina) were used for averaging the gene expression of multiple probes, corresponding to a unique Entrez gene ID within a microarray. In this way, probes unique for a specific microarray platform or even for a specific platform version were omitted. On the basis of 48,804 probes (representing more than 47,000 gene transcripts) on the Illumina microarray [20], [21] and 54,120 probe spots (representing over 47,000 gene transcripts of approximately 38,500 genes) on the Affymetrix microarray [22], our merged and aggregated dataset consisted of 10,286 genes with unique Entrez gene IDs. Data pre-processing and subsequent statistical analyses were performed in the R statistical environment (version 2.12.0) [23] using affy [24], lumi [25] and limma [26] software packages. To find differentially-expressed genes (DEGs), transriptomics data of cancer and non-cancer samples (referred to as control) were compared. Ranks (and rank products) of gene expression values were taken into analysis instead of the actual gene expression values, by using a Bioconductor package *RankProd* [27]. Rank product was calculated for the rank of each gene list divided by the number of probes (Entrez IDs) of each microarray. Comparing them with permuted files allowed the determination of statistical significances of difference between DEGs. Two classes of the data (the cancer and normal samples) were specified during the ranking procedure. After ranking, (down- and upregulated) DEGs were extracted and searched for intersection (by unique Entrez IDs) with a list of human proteases (669) and protease inhibitors (242) obtained from MEROPS, the peptidase database (http://merops.sanger.ac.uk/) [11] selected on 04/18/2011 for known and putative proteases and on 12/20/2011 for protease inhibitors (Files S1 and S2). The analysis pipeline is shown in Fig. 1A.

Five sublists of differentially-expressed protease and protease inhibitor genes in GBM tissues and cell lines were compared (tissue samples results and cell lines results from cross-platform and same-platform comparison). Venn diagrams were used to search for jointly upregulated/downregulated genes in GBM tissue and GBM cell lines with a percentage of false prediction (PFP) <0.05. Venn diagrams were drawn separately for the 2 GBM cell lines, the protease and protease inhibitor genes and up- and downregulated genes.

The criteria for candidate gene selection were set as: (1) the genes are overexpressed in GBM tumors tissues and in at least one GBM cell line (U87-MG or U373), because it can then be assumed that the gene is overexpressed in cancer cells in GBM tumor tissue and not in the microenvironment, enabling further *in vitro* studies using GBM cell lines); (2) the genes are upregulated in both our experimental data sets and publicly-available data sets (and therefore after cross- and same-platform comparison) and (3) their PFP should be <0.05.

### Validation of transriptomics data

#### Cell culture and tissue sample collection

Culture of human GBM cells (U87-MG, U373) is described above. NHA cells were purchased from Lonza (Slough, UK) and cultured in low-glucose DMEM medium (Sigma-Aldrich, St. Louis, MO, USA) supplemented with 10% fetal bovine serum (FBS; PAA, Pasching, Austria), 100 U penicillin/streptomycin (PAA), 2 mM L-glutamine (PAA) and 20 nM HEPES buffer solution (Gibco, Life Technologies). U87-MG cells of passages 29, 30 and 31, U373 cells of passages 40, 41 and 42, and NHA cells of passages 2 and 6 were used in the experiments.

Frozen tissue samples from GBM patients were obtained from the Cancer Center Amsterdam, VU University Medical Center Amsterdam, whereas frozen control post-mortem non-malignant brain tissue samples were obtained from the Academic Medical Center of the University of Amsterdam. Local ethics committees waved use of material, as it fell under the Dutch Code of proper secondary use of human tissue. The research was performed on ‘waste’ material, stored in a coded fashion.

#### Real time qPCR (RT-qPCR) analysis

mRNA was extracted from 3 biological replicates of each cultured cell type (U87-MG, U373 and NHA) using Trizol reagent (Invitrogen, Life Technologies) and its quality and concentration were determined by NanoDrop NT-1000 (Thermo Fisher Scientific, Waltham, MA, USA). cDNA was synthesized using High Capacity cDNA Reverse Transcription Kit (Applied Biosystems) and Gene AMP PCR System 9700 (Applied Biosystems). Gene expression (RT-qPCR) analyses were performed using TaqMan Gene Expression Assays (Applied Biosystems): Hs00951083_m1 (for *TFRC*), Hs00166156_m1 (for *CTSK*), Hs01049561_m1 (for *GFPT2*), Hs01073631_m1 (for *ERAP2*), Hs00173754_m1 (for *GPR56*), Hs00269961_m1 (for *CD74*), Hs00160066_m1 (for *PI3*), and human GAPD (*GAPDH*) as a reference (No.: 4310884E) and ABI Prism 7900 Sequence Detection System (Applied Biosystems).

Data analysis was performed using the standard curve method [28] and transcripts of interest were normalized using reference genes.

RT-qPCR analysis of *CTSK* mRNA was repeated using both GBM cell lines (U87-MG and U373; 3 biological repetitions of each), GBM tissues (8 samples), commercially-available normal brain pooled mRNA (FirstChoice Human Brain Reference RNA; 23 pooled samples; Applied Biosystems) and commercially-available NHA mRNA (human astrocyte total RNA; ScienCell Research Laboratories, Carlsbad, CA, USA) with the geometrical mean of *TBP* (assay number Hs00427620_m1) and *HPRT1* (assay number Hs02800695_m1) mRNA as a reference [29]. Data analysis was performed in the same manner as described above.

#### Functional characterization of candidate genes

Candidate genes were queried by Biomine [30] search engine (http://biomine.cs.helsinki.fi/search/), which integrates cross references from several biological databases into a graph model with multiple edges corresponding to protein interactions, gene-disease associations and gene-ontology annotations. With the use of this approach, process and inter-candidate genes' linkage were revealed and were applied for prediction of a biologically-relevant role in GBM.

Since we were interested in novel functions of the gene products that were upregulated in both GBM tissues and cells, a literature search was performed to create a scheme of candidate genes that are relevant for cancer progression.

#### Western blot

Proteins were extracted from tissues and cells using Cell Lysis Buffer #9803 (Cell Signaling Technology, Danvers, MA, USA), pH 7.5, and supplemented with 10 µM pepstatin A and 0.5 mM PMSF following the manufacturer's instructions. Conditioned media of GBM cell lines (their growth media) were collected after 24 h incubation, concentrated using lyophilization and supplemented with pepstatin A and PMSF in the same manner as tissue and cell lysates. Due to high instability of isolated active CatK [31], protein extracts were directly subjected (without measuring the concentration) to SDS/PAGE electrophoresis with 12% polyacrylamide gel for 1.5 h at 12 mA and 1.5 h at 30 mA. Proteins bandings were transferred to Immun-Blot PVDF Membrane (Bio-Rad, Hercules, CA, USA) overnight at 16 V and 4°C.

After transfer, the membranes were blocked with Blotting-Grade Blocker (Bio-Rad) diluted in 0.1% Tween in 1× PBS, the buffer that was also used for antibody dilutions, and probed with rabbit polyclonal anti-CatK antibody ab19027 (Abcam, Cambridge, UK; dilution 1∶2000) overnight at 4°C. Anti-β-actin rabbit polyclonal primary antibody ab8227 (Abcam; dilution 1∶5000) was used to detect actin as loading control. The anti-rabbit secondary antibody W401B conjugated with horseradish peroxidase (Promega, Madison, WI, USA; dilution 1∶2500) was applied for 1 h at room temperature. In between, membranes were washed with 0.1% Tween in PBS. Chemiluminescence detection was achieved using the Amersham ECL detection system (GE Healthcare, Chalfont St. Giles, Buckinghamshire, UK) according to the manufacturer's instructions. As a control for cathepsin K expression, recombinant pro- and active CatK were used (kindly provided by Dr. Marko Novinec from Faculty of Chemistry and Chemical Technology of the University of Ljubljana, Slovenia).

Additional experiments have been performed using cell lines U87-MG and U373 from which the proteins were extracted by using lysis buffer solutions (50 mM Tris, 30% Brij 35, 20 mM DTT, 5 mM EDTA; pH = 6.9) with the addition of various protease inhibitors and concentrations were measured. The following inhibitors were used: E-64 (inhibitor of cysteine proteases), CA074 (inhibitor of CatB), CLIK148 (inhibitor of CatL) and pepstatin A (inhibitor of aspartic proteases) at 5 µM concentration, PMSF (inhibitor of serine proteases) at 1 mM concentration, EDTA (inhibitor of metalloproteases) at 20 mM concentration and a combination of all of them. The concentration of proteins loaded was 20 µg for U87-MG cells and 8 µg of U373 cells.

#### Immunohistochemistry and immunocytochemistry

GBM cryostat sections (8-µm thick) of 8 patients and unmatched non-malignant brain tissue sections of 7 individuals (4 from the hippocampus and 3 from the temporal cortex) were used for immunohistochemistry (IHC). Sections were blocked with 10% goat serum (Dako, Glostrup, Denmark) in PBS and stained with rabbit polyclonal anti-CatK antibody ab19027 (Abcam; the same as for western blots, based on the manufacturer's information on the application in both techniques) at a 1∶200 dilution in PBS containing 1% FBS overnight at 4°C followed by 1× PBS wash and an incubation with goat anti-rabbit secondary antibody conjugated to horseradish peroxidase (Dako) at a 1∶200 dilution.

Detection was performed using the AEC substrate kit to detect peroxidase activity (Vector Laboratories, Burlingame, CA, USA).

Upon IHC, samples were counterstained using hematoxylin and covered using glycerin-gelatin jelly. Paraffin sections of mouse jaw (5-µm thick; kindly provided by Mr. Ton Schoenmaker from the Academisch Centrum Tandheelkunde Amsterdam (ACTA), The Netherlands) with osteoclasts were used as positive control. Staining of sections was analyzed with the use of a Vanox-T photomicroscope with a 10× objective (Olympus, Tokyo, Japan) and a CFW-1312M 1360×1024 pixel 10-bit monochrome FireWire camera (Scion, Tucson, AZ) mounted on the front part of the Vanox microscope using adapting optics according to Chieco et al. [32].

The same protocol was used for immunocytochemistry (ICC) using U87-MG cells (passage 35) and U373 cells (passage 47) grown on the Lab-Tek Chamber Slide System (Thermo Scientific, Waltham, MA, USA) and on 96-well plates coated with poly-L-lysine (Sigma-Aldrich). Osteoclasts from peripheral blood were used as positive control (grown on 96-well plates) and kindly provided by Mr. Ton Schoenmaker and Ms. Ineke Jansen (ACTA).

## Results

### Transriptomic analyses reveal differentially-expressed genes of proteases and protease inhibitors in GBM tissues and cell lines

Two lists of DEGs with PFP <0.05 for each cell line (Illumina versus Affymetrix and Affymetrix versus Affymetrix) and one list for tissue samples (Affymetrix versus Affymetrix) were generated, i.e. in total 5 lists of DEGs (File S3; upregulated and downregulated genes are listed separately). A subset of differentially-expressed protease and protease inhibitor genes was obtained from the intersection of lists of DEGs with our predefined list of genes (378 genes were included from the MEROPS database, of which 311 were protease genes and 75 were protease inhibitor genes. In total, of the 10,286 genes in the integrated microarray datasets, 378 genes were differentially expressed. Of those, 8 genes overlapped with genes from the MEROPS database. File S4 shows the lists of genes in the integrated microarray datasets, and File S5 shows the list of genes extracted from these lists.

**Figure 2:**
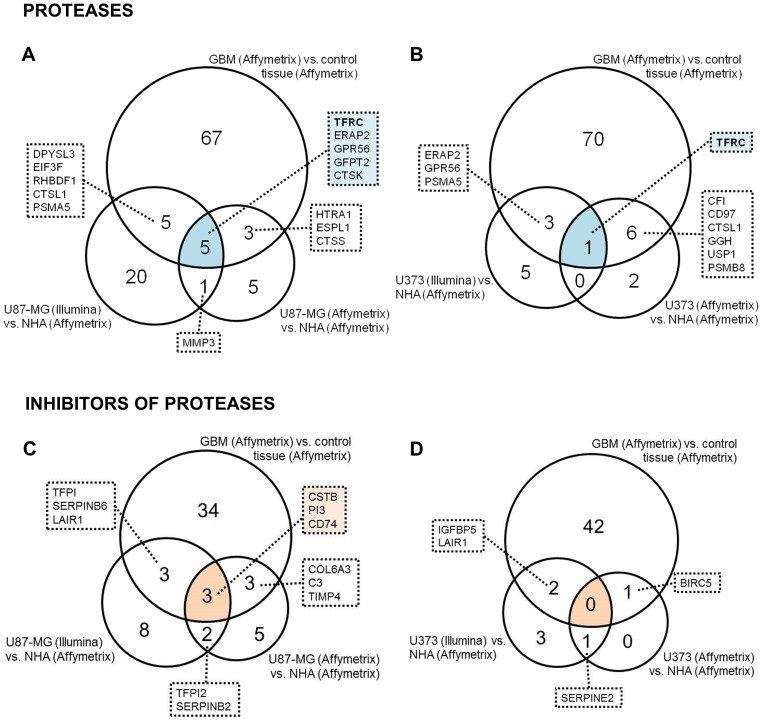
Venn diagrams of protease and protease inhibitor genes upregulated in both GBM tissue and GBM cells. In total, 669 protease and 242 protease inhibitor genes were checked for deregulation in GBM tissues and cells in comparison to non-malignant brain tissue and NHA cells. Venn diagrams include upregulated genes in GBM tissues and cells with PFP >0.05. Candidate genes appear in the intersections of Venn diagrams. Comparisons of protease genes upregulated in GBM tissue and in U87-MG cells (A) and upregulated in GBM tissue and in U373 cells (B) across Illumina and Affymetrx platforms and within the Affymetrix platform only are shown. The other 2 Venn diagrams show comparisons of protease inhibitor genes upregulated in GBM and in U87-MG cells (C) and upregulated in GBM and in U373 cells (D) across Illumina and Affymetrx platforms and within the Affymetrix platform only.

Differentially-expressed protease and protease inhibitor genes (Files S6 and S7) differed both in their number and identity when comparing GBM tissue and cell lines, as shown in the heatmap (Fig. 1B), implying discrepancies between gene expression in tissues and cell lines and between different cell lines. Cross-platform comparison (Illumina versus Affymetrix) gave different results in comparison with same-platform comparison (Affymetrix versus Affymetrix). In case of protease inhibitors U87-MG and U373 cell lines clustered together independently from microarray technology. Five protease genes (Fig. 2A,B), and 3 protease inhibitor genes (Fig. 2C,D) appeared in the intersection of the Venn diagrams of overexpressed genes (drawn on the basis of all lists of upregulated protease and protease inhibitor genes) and matched the criteria for candidate gene selection as is described in the Data analysis section. These criteria were valid for 2 metalloprotease homologous genes: transferrin receptor (*TFRC*) and endoplasmic reticulum aminopeptidase 2 (*ERAP2*), a serine protease homologous G-protein-coupled receptor 56 (*GPR56*), and two genes with homology to cysteine proteases: the glutamine-fructose-6-phosphate transaminase 2 (*GFPT2*) and cathepsin K (*CTSK*). The 3 inhibitor genes identified were stefin B (*CSTB*), peptidase inhibitor 3 (*PI3*; also called elafin) and CD74 (*CD74*; MHC class II chaperone). The *TFRC* gene was the only gene found to be overexpressed in GBM tissue and in both selected GBM cell lines, across both microarray platforms.

The same analysis was performed with downregulated protease and protease inhibitor genes which in contrast revealed only one protease gene that matched our criteria, the serine protease carboxypeptidase E (*CPE*) (Fig. S1).

### Validation of transcriptomic analysis results with RT-qPCR

**Figure 3:**
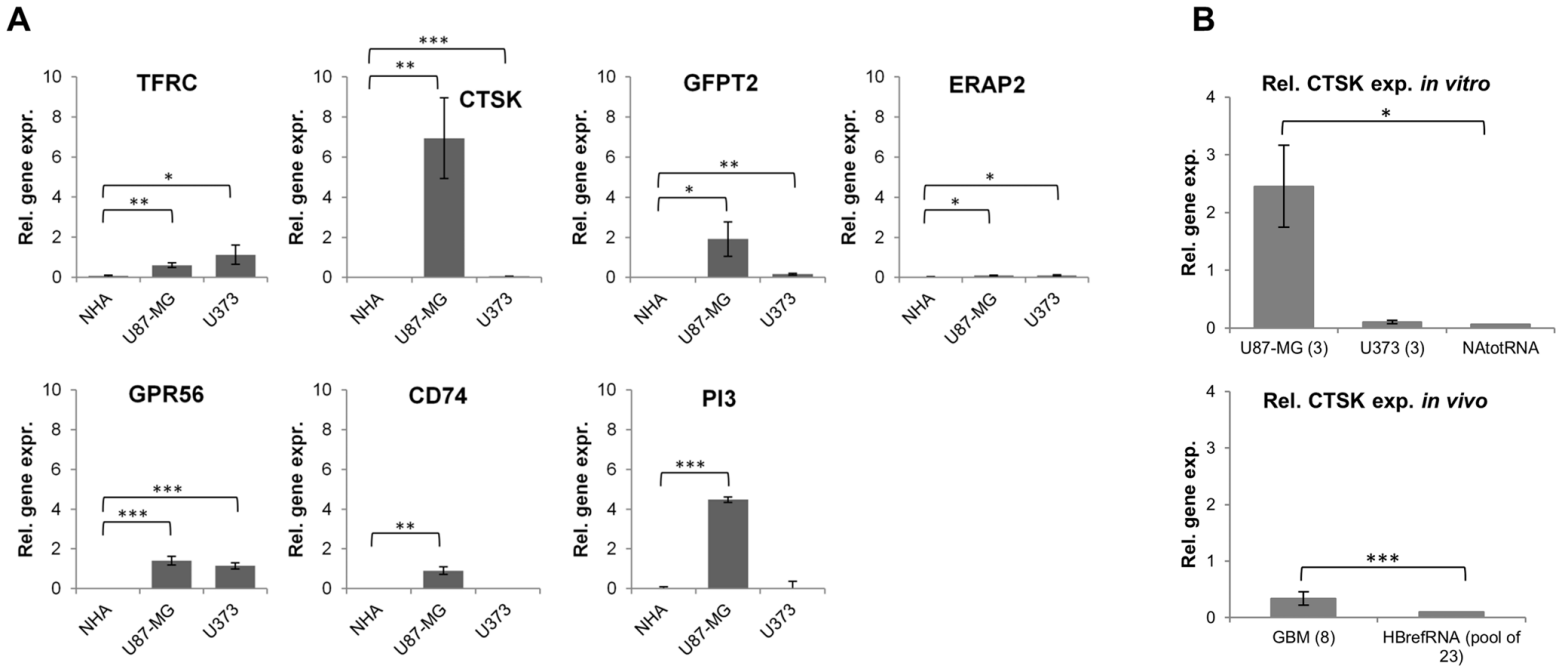
RT-qPCR analysis of expression of selected proteases and protease inhibitors in U87-MG and U373 GBM cells, NHA cells and GBM tissues and non-malignant brain *(in vivo)*. (A) Upregulated expression of seven genes (*TFRC*, *CTSK*, *GFPT2*, *ERAP2*, *GPR56*, *CD74*, *PI3*) as determined by microarray data was validated in GBM cells in comparison to NHA cells by RT-qPCR, using GAPDH as reference gene. (B) Additional RT-qPCR analysis of expression of the *CTSK* gene using GBM tissues and cell lines with reference genes *TBP* and *HPRT1* in comparison to NHA cells (NAtotRNA) and non-malignant brain (HBrefRNA). The experiments were performed in triplicate (except for 8 repetitions of GBM tissue and commercial RNA from NHA and normal brain, used in experiment B). Error bars represent standard deviation; * p-value<0.05, ** p-value<0.01, *** p-value<0.001.

RT-qPCR analyses of the GBM cell lines U87-MG, U373 and control NHA cell line confirmed overexpression of all seven candidate genes (*TFRC*, *GFPT2*, *ERAP2*, *GPR56*, *CTSK*, *CD74* and *PI3*) in GBM cell lines (Fig. 3A). Among these, the *CTSK* gene was found to be more than 1000-fold overexpressed in U87-MG cells and more than 10 times overexpressed in U373 cells, when compared to NHA cells. Both protease inhibitor genes (*CD74* and *PI3*) were overexpressed only in U87-MG cells. *CD74* and *PI3* genes were found to be 4×10^4^ times and 2×10^5^ times overexpressed in U87-MG cells in comparison to NHA cells.

Additional RT-qPCR analysis of expression of the *CTSK* gene and different reference genes in both GBM cell lines and GBM tissues in comparison to the NHA cell line and control brain tissue, respectively, were in line with our observations that the *CTSK* gene is overexpressed in GBM cell lines. *CTSK* overexpression was significant in GBM tissues as well (Fig. 3B). Boxplots of Ct values of reference genes, are shown in Fig. S2.

### Biomine query and literature search of selected candidate genes

**Figure 4:**
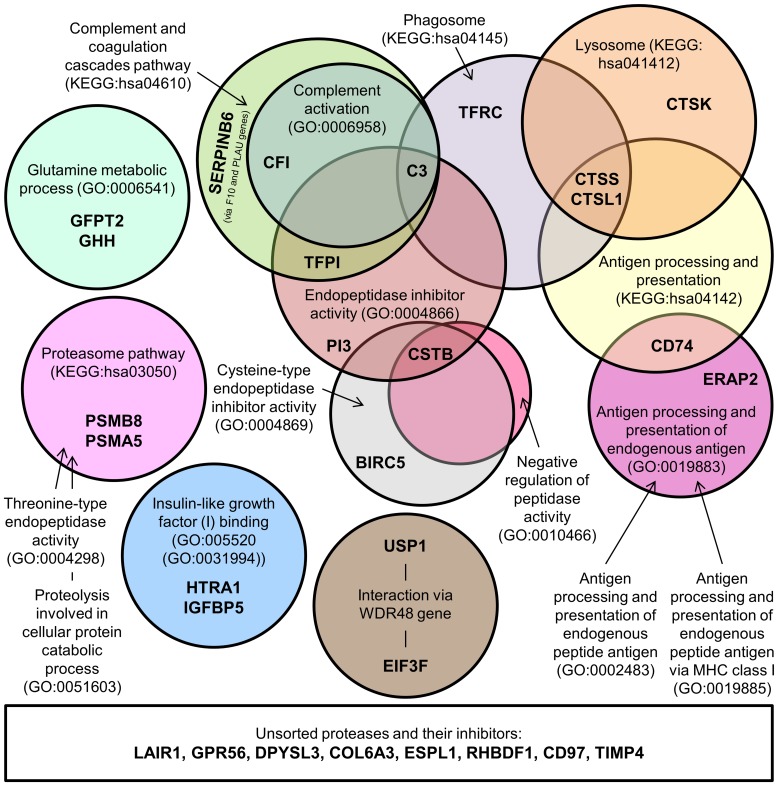
Scheme of cellular processes and activities involving overexpressed protease and protease inhibitor genes in GBM. The overexpressed protease and inhibitor genes in GBM tissues and cells were queried by the Biomine search engine which identified processes and activities ascribed with KEGG and GO identifiers (in circles) in which selected genes (in bold caption) are involved.

Biomine query of the overexpressed protease and protease inhibitor genes in GBM tissues and in GBM cell lines revealed KEGG and GO identifiers of processes, in which these genes are involved (Fig. 4). These processes varied from receptor (IGF1) binding to catabolic phagosomal and lysosomal processes and antigen processing.

**Figure 5:**
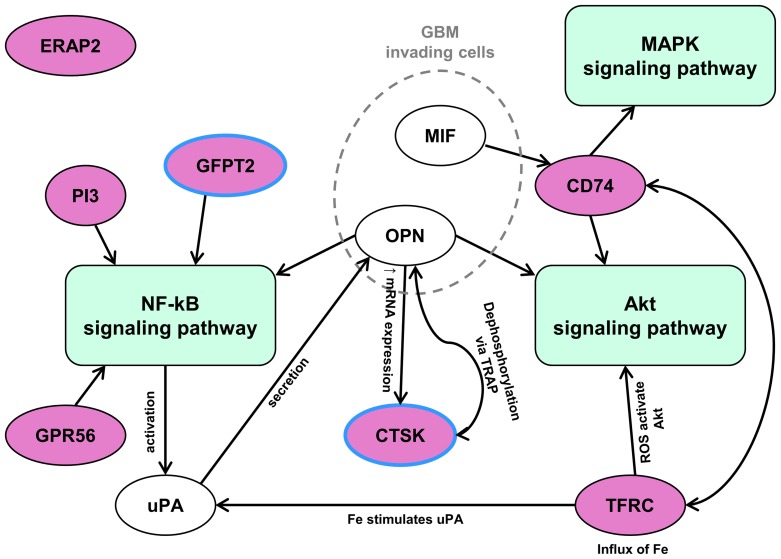
Signaling pathways in which the candidate genes are involved. An extensive literature search revealed that the candidate genes are cross-linked to 3 signaling pathways: NF-κB, Akt and MAPK, which all play a role in cancer. NF-κB signaling pathway has a crucial role in regulating immune responses, whereas Akt signaling has been shown to inhibit the growth of GBM cells and GBM stem-like cells that may also be impaired by MAPK signaling disruption. Because of the RT-qPCR results, *CTSK*'s role has been examined and it was found via cross linking to other candidate genes obtained via osteopontin (*OPN*) gene functions.

An additional literature search enabled us to generate a scheme of the involvement of the candidate genes in signaling pathways relevant for cancer progression (Fig. 5). The results of this analysis show that most candidate genes are associated with 3 signaling pathways, the NF-κB (KEGG: hsa04064), PI3K-Akt (KEGG: hsa04151) and MAPK (KEGG: hsa04010) pathways, which all have a role in cancer, implying that our candidate gene selection method is relevant to identify protease and protease inhibitor genes that are crucial for GBM pathology.

Only 2 of the 7 candidate genes, *GPR56* and *CTSK*, have so far not been associated with GBM. *CTSK*, coding for cysteine protease cathepsin K (CatK), has been selected for further molecular validation because of its high upregulation. One of the known physiological role of CatK is its involvement in bone resorption. We found cross-linkage with other candidate genes via the added osteopontin (*OPN*) gene. *OPN* has been reported to be overexpressed in GBM as well [15].

### Cathepsin K validation at the protein level

**Figure 6:**
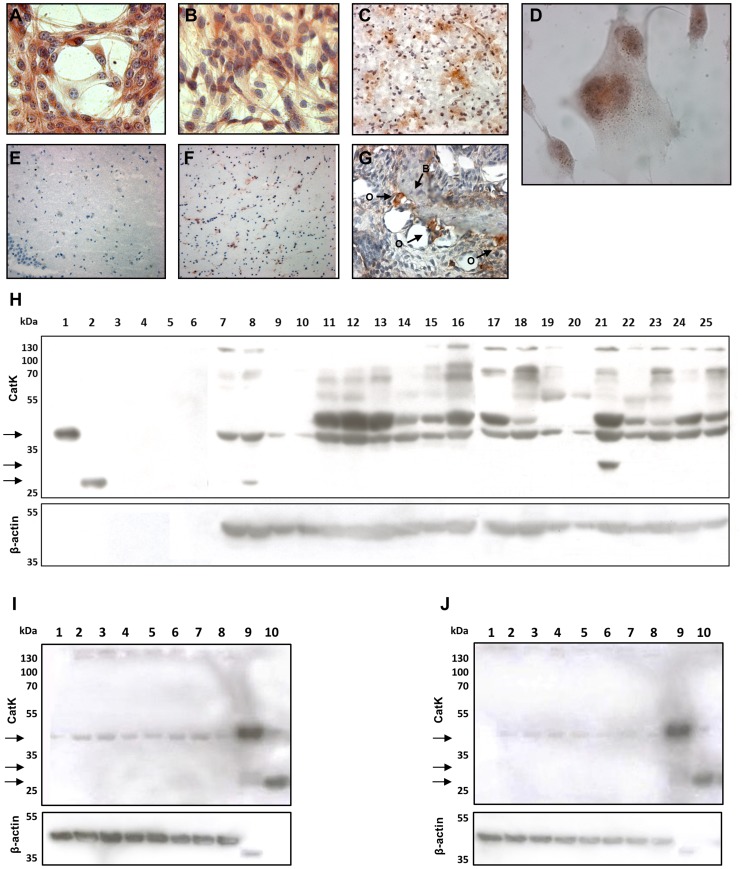
Immunohistochemstry and immunocytochemstry and Western blot analysis of cathepsin K in GBM cells and tissues. ICC staining of CatK in U87-MG (A) and U373 (B, D) GBM cells. At high magnification (1000×), a granular pattern of the staining was observed in U373 cells (D), which corresponds to perinuclear endo-lysosomal-like localization of CatK. Strong IHC staining of CatK in GBM tissue (C), and weak staining in control brain tissue (non-malignant brain) (E, F). Mouse jaw with bone tissue (B) with osteoclasts at bone edges (O) was used as positive control for CatK staining (G). Magnifications: A, B, G - 400×; D - 1000×; C, E, F - 200×. (H) Western blot of GBM cells (lanes 7–10) and their conditioned media (CM; lanes 3–6), GBM tissue (lanes 17–25) and non-malignant brain tissue (lanes 11–16) showing positivity for pro-CatK (39 kDa). The active form of the enzyme (27 kDa) was detected only in a small amount in one passage of U87-MG cells (lane 8). At 30 kDa, an intermediate form of CatK was observed (lane 21). Recombinant proform (lane 1) and active CatK (lane 2) were used as positive control. As loading control β-actin was used. Legend: 1 – recombinant pro-CatK, 2 – recombinant active CatK, 3 – U87p38 CM, 4 – U87p39 CM, 5 – U373p41 CM, 6 – U373p42 CM, 7 – U87p45, 8 – U87p48, 9 – U373p45, 10 – U373p48, 11–16 – different non-malignant brain samples, 17–25 – different GBM tissue samples. **Please note** that Western blotting image does not allow for direct quantitative comparison of CatK expression in control and tumor samples due to variable protein amounts loaded. (I and J) Additional western blot experiments using cell lines U87-MG (I) and U373 (J) and different protease inhibitors. No active form of cathepsin K was observed in any of the conditions tested but in all cases pro-cathepsin K was present. Legend: 1 – without any inhibitor, 2 – 5 µM E-64, 3 – 5 µM CA074, 4 – 5 µM CLIK148, 5 – 5 µM pepstatin A, 6 – 1 mM PMSF, 7 – 20 mM EDTA, 8 – combination of all inhibitors, 9 – control: recombinant pro-CatK, 10 – control: recombinant active CatK.

IHC and ICC were used to determine (sub-)cellular and tissue distribution patterns of CatK protein (Fig. 6). In the cultured GBM cells, staining of the CatK protein was observed in perinuclear area of cells (Fig. 6D). Cryostat sections of 8 GBM samples all stained strongly for CatK, displaying a pattern of high intensity staining around blood vessels. Staining of osteoclasts was taken as positive control (Fig. 6G). Non-malignant brain tissue either did not stain at all for Cat K protein (Fig. 6E) or stained only weakly for CatK (Fig. 6F). In particular, 2 out of 4 hippocampus samples and 2 out of 3 temporal cortex samples showed positive staining (that was considerably weaker than in GBM samples). Staining of Cat K protein suggested that CatK in GBM is not localized at invasive tumor edges.

The controls for IHC and ICC staining using the Cat K antibody are shown in Fig. S3.

Western blotting confirmed CatK expression at the protein level in GBM tissue, non-malignant brain tissue and GBM cells but not in conditioned media collected from GBM cells (Fig. 6H). It also confirmed the specificity of the anti-cathepsin K antibody previously used in IHC and ICC experiments. Western blots of extracts of GBM cells and tissues and non-malignant brain tissues revealed the presence of pro-CatK (39 kDa) in all samples examined, whereas the active CatK (27 kDa) was not detected in any of the samples tested except for one passage of U87-MG cells (lane 8). Only in one GBM tissue sample (lane 21) an intermediate form of CatK was observed at 30 kDa, suggesting that only the proteolytically-inactive proform of CatK is overexpressed. Furthermore, multiple bands of higher molecular mass were persistently detected and highly labelled particularly in the tissue samples (lanes 11–25). Additional western blot experiments using protease inhibitors that selectively inhibit the 4 classes of proteases (Fig. 6I for U87-MG cells and Fig. 6J for U373 cells), did not give any evidence of proteolytic processing of the CatK proform in the GBM cell lines tested, as no band at 27 kDa was detected in any of the cell extracts.

## Discussion

The aim of our study was to identify proteases and protease inhibitors, which are overexpressed in GBM in comparison to non-malignant brain. These proteases may represent candidate biomarkers related to prognosis and response to therapy. We found that the expression of these genes differ between microarray platforms (Illumina Bead Chip versus Affimetrix GeneChip). This affects the selection of candidate genes for biological validation, especially when only one microarray platform is used (which generally is the case). Gene expression data, including data on cancer in comparison to control tissue, are becoming publicly available, and their use to address specific research questions has become an interesting opportunity for *in silico* analyses [14]. It allows for increased statistical power of gene expression analysis. Special attention has to be given to the way datasets are merged and the meta-analysis is carried out in order to take advantage of the higher statistical power. Several approaches are possible to combine information from multiple microarray studies as is summarized by Scharpf et al. [33]. Raw data were taken and quality control and pre-processing were performed seperately for each of the datasets in the meta-analysis.

Since expression values from different platforms are not comparable per se, we used ranks and rank products of gene expression by using *RankProd* library [27] to obtain reliable results on elevated protease and protease inhibitors gene transcripts.

Tumors are heterogeneous masses of cells, comprising of cancer cells and various types of non-cancer cells such as stromal cells, inflammatory cells, blood vessel cells, all of which may express a variety of proteases and protease inhibitors. Therefore, we have identified expression of the genes that are overexpressed in GBM tissue samples as well as in GBM cancer cells only versus their control, physiological counterparts, such as non-malignant brain tissue and human astrocytes, respectively. In addition, GBM tissue and GBM cell line cultures were used for their validations. Two commercially-available GBM cell lines U87-MG and U373 were selected, which differ in a number of properties including invasion, the U373 cells being more invasive than the U87-MG cells [18]. Here, we demonstrate that only approximately 5% of overexpressed protease and protease inhibitor genes in tumor tissue were overexpressed in the GBM cell lines. It remains to be elucidated whether these differences are due to the other types of cells present in the tumor besides cancer cells, or are due to interactions between the different cell types affecting the GBM proteolytic profile. It is becoming increasingly apparent that crosstalk between cancer cells and cells of the tumor stroma (the microenvironment) is involved in invasive growth and metastasis [34]. It implies that phenotypes of GBM do not arise only in a strictly cancer cell-autonomous manner and that the heterogeneity of tumor mass needs to be considered in all aspects of cancer research. As a consequence, their manifestation of cancer cell phenotypes cannot be understood solely through analyses of the cancer cell genomes. Our results demonstrate that U87-MG and U373 cell lines poorly resemble GBM tissue with respect to changes observed in protease and protease inhibitor gene expression, most likely because of the tumor microenvironment.

Here, we report the most robust set of upregulated protease and protease inhibitor genes, which were jointly expressed in GBM tumor tissue and in at least one GBM cell line, i.e. the protease genes *TFRC*, *GFPT2*, *ERAP2*, *GPR56* and *CTSK*, and the protease inhibitor genes *CSTB*, *CD74* and *PI3*. The proteins, coded by those genes as listed by KEGG and GO identifiers, are involved in antigen processing and presentation (*ERAP2* and *CD74*), lysosomal peptidase activity and its regulation (*CTSK*, *PI3* and *CSTB*), glutamine metabolism (*GFPT2*) and phagosome formation (*TFRC*). These activities were linked to 3 signaling pathways with significant roles in cancer: NF-κB, PI3K-Akt and MAPK [35]. Most of the genes have been reported to be linked to cancer and/or GBM progression.

Only one gene matched our criteria for downregulated expression, namely *CPE*. *CPE* may well be relevant for GBM because upregulated CPE levels are associated with the proliferation rate of glioma cell lines and downregulated CPE levels are associated with migration and invasion of the cells [36]. We did not investigate the downregulated expression of *CPE* any further because the aim of our study was to identify overexpressed genes in both GBM tissues and cells because of the greater relevance for clinical applications.

Overexpression of transferrin receptor (*TFRC*) is not surprising, as the protein is responsible for binding transferrin and intracellular uptake of iron. High levels of iron are needed in GBM to meet energy requirements associated with rapid growth and this system itself creates a positive feedback mechanism in which high amounts of ROS and *TFRC* perpetually induce each other and drive GBM proliferation. Moreover, glutamate release from cancer cells is mediated by *TFRC* which makes GBM cells exotoxic for neurons and provides space for the progression of tumor mass [2], [35]. Therefore, our study supports that *TFRC* is a potential target for glioma therapy [37].

G protein-coupled receptor 56 (*GPR56*) is involved in adhesion signaling. It is expressed at the leading edge of filopodia and co-localizes with α-actin. *GPR56* is also a stem cell marker and was proposed as therapeutic target [38].

Endoplasmic reticulum aminopeptidase 2 (*ERAP2*) plays a central role in peptide trimming, a step required for the generation of most HLA class I-binding peptides. Altered expression of the *ERAP2* gene may favor escape of cancer cells from immune surveillance [39].

Glutamine-froctose-6-phosphate transaminase 2 (*GFPT2*) controls the flux of glucose into the hexosamine pathway and is most likely involved in regulating the availability of precursors for N- and O-linked glycosylation of proteins [40].

With respect to the inhibitors, we found most interesting that expression of the cysteine protease inhibitor gene of the stefin B (cystatin B; *CSTB*) was upregulated. This intracellular inhibitor is widely distributed in many cell types, and belongs to the cystatin superfamily, presumably balancing the intracellular activities of lysosomal cysteine cathepsins [12]. One would expect downregulation rather than upregulation in cancer, because CatB, CatL and CatS have been found to be upregulated in glioma [3], and their expression was found to be correlated with malignancy [4], [6], [8], [41]. However, stefin B has also been found to be upregulated and secreted in other types of cancers, which indicates other functions of this protease inhibitor [42]. Recently, Sun et al. [43] showed increased expression of stefin B in the nucleus of T98G astrocytoma cells that was associated with delay in cell cycle progression and stefin B was also found to inactivate caspase-3/7 in the nucleus. On the other hand, Gole et al. [44] reported that expression of stefin B is decreased in invading GBM cells and at the periphery of the tumor in orthotopic xenografts of GBM in nude mice indicating differential expression of stefin B in different subpopulations of GBM cells within a tumor.

Peptidase inhibitor 3 (*PI3*) correlates with poor survival of GBM patients and has been proposed as prognostic marker and therapeutic target [45]. *PI3* is an elastase inhibitor and plays a central role in the control of neutrophil elastase activity, the latter not being expressed in GBM cells. However, infiltrated neutrophils seem to may be relevant part of the GBM microenvironment and tumor progression and even prognosis [46].

*CD74* is a MHC class II chaperone, acting as membrane receptor for the pro-inflammatory cytokine macrophage migration inhibitory factor and promotes cell proliferation and survival. *CD74* overexpression has been observed in several non-central nervous system cancers, where it was associated with aggressive behavior and poor prognosis, whereas in GBM its potential role in temozolomide resistance has been reported [47].

Cysteine cathepsins have been extensively investigated in glioma by our research groups [4], [6], [8], [41], [44], [48] and by others [3], [10]. CatB, CatL and CatS have been reported to be associated with glioma progression, and are highly overexpressed at mRNA, protein and activity levels in GBM. CatB and CatS [48]–[50] have also been associated with invasion and patient survival whereas CatL is most likely involved in transcription factor activation in apoptosis and cell proliferation [6], [8], [43]. However, our protease expression study indicates that CatK is much more overexpressed in GBM. *CTSK* that encodes for CatK may be the most interesting candidate gene that has never been associated with GBM, in contrast to other cysteine cathepsins [6], [44]. CatK is involved in collagenolytic activity in relationship with bone remodeling and is highly expressed in osteoclasts [48], [49]. Because of its involvement in pycnodysostosis [51], osteoporosis [52], [53] and invasive carcinomas and their metastasis, a series of small inhibitory compounds have been developed for clinical application [53]. CatK knockdown animals show considerable neural defects indicating its role in normal brain functioning [54], [55], which is in contrast to CatB or CatL-deficient animals [56], although in CatB^−/−^ and CatL^−/−^ double knock-out mice, brain atrophy due to massive apoptosis of neurons is lethal [52]. In astroglia-rich primary cultures from CatK-deficient mice it appeared that CatK plays a role in differentiation of oligodendrocytes [52]. Recent studies revealed CatK expression in many more cell types than osteoclasts [57]–[61]. It is likely that CatK cleaves other physiological substrates besides its unique cleavages of collagen fibrils.

This study is the first report of overexpression of CatK in GBM tissues and cells at the mRNA and protein level. In both GBM cell lines, CatK immunostaining was localized perinuclearly, likely in endo-lysosomes, whereas in GBM tissues staining was found mainly around blood vessels. In GBM tissue and cells, CatK was mostly present in precursor forms of 39 and 30 kDa indicating a non-proteolytic function of the proform. In contrast to other cathepsins [6], [10], the proform of CatK was not secreted, being absent in conditioned media from GBM cells (Fig. 6F). Western blotting revealed higher molecular mass bands that were positive for CatK and may represent complexes of CatK and glycosaminoglycans, such as chondroitin sulfate, which are primary components of the ECM in brain. Chondroitin sulfate promotes collagenase activity of CatK by supporting auto-processing of the pro-domain of CatK in an acidic environment [62]–[64]. The higher molecular mass bands may also represent polymeric complexes of CatK that facilitate collagen degradation [65].

To conclude, we applied bioinformatics and statistics tools to GBM tissues and cells to identify novel protease and protease inhibitor genes that are overexpressed in malignant cells of GBM. The analysis revealed the highest overexpression of CatK in GBM at the mRNA and protein levels, indicating a functional role in GBM progression that remains unknown. Future studies will be focused on what proteins CatK cleave in these tumors and on the evaluation of its role in GBM progression.

## Supporting Information

Figure S1 **Venn diagrams of protease and protease inhibitor genes downregulated in both GBM tissue and GBM cells.** In total, 669 protease and 242 protease inhibitor genes were checked for deregulation in GBM tissues and cells in comparison to non-malignant brain tissue and NHA cells. Venn diagrams include downregulated genes in GBM tissues and cells with PFP >0.05. Candidate genes appear in the intersections of Venn diagrams. Comparisons of protease genes downregulated in GBM tissue and in U87-MG cells (A) and downregulated in GBM tissue and in U373 cells (B) across Illumina and Affymetrx platforms and within the Affymetrix platform only are shown. The other 2 Venn diagrams show comparisons of protease inhibitor genes downregulated in GBM and in U87-MG cells (C) and downregulated in GBM and in U373 cells (D) across Illumina and Affymetrx platforms and within the Affymetrix platform only. Only one protease gene matched our selection criteria, *CPE* coding for carboxypeptidase E.
(TIF)

Figure S2 **Boxplots of Ct values of reference genes (GAPDH, HPRT1 and TBP) used for RT-qPCR analysis.** Median values are shown with box limits indicating the 25th and 75th percentiles as determined by R software; whiskers extend 1.5 times the interquartile range from the 25th and 75th percentiles and outliers are represented by dots. Sample points (biological replicates×technical replicates: n): A) n_U87_MG_ = 6, n_U373_ = 6, n_NHA_ = 6; B and C) n_U87_MG_ = 6, n_U373_ = 6, n_NAtotRNA_ = 2, n_GBM_ = 16, n_HBrefRNA_ = 2.
(TIF)

Figure S3 **Immunohistochemical and immunocytochemical control staining.** CatK staining was performed in the presence (B,D,F) or absence (A,C,E) of primary anti-CatK antibody. (A and B) osteoclasts in culture; (C and D) U373 cell line; (E and F) GBM tissue section. Magnifications: A–D, 200×; E–F, 100×.
(TIF)

File S1 **List of all known and putative MEROPS proteases.**
(XLS)

File S2 **List of all known and putative MEROPS proteases inhibitors.**
(XLS)

File S3 **Differentially-expressed protease and protease inhibitor genes.**
(XLS)

File S4 **Protease and protease inhibitor genes in the integrated microarray.**
(XLS)

File S5 **Protease and protease inhibitor genes that were selected for further analyses.**
(XLS)

File S6 **Selected differentially-expressed protease genes.**
(XLS)

File S7 **Selected differentially-expresses protease inhibitor genes.**
(XLS)
