# Correction: Expression Analysis of All Protease Genes Reveals Cathepsin K to Be Overexpressed in Glioblastoma

**DOI:** 10.1371/journal.pone.0142116

**Published:** 2015-10-29

**Authors:** Urška Verbovšek, Helena Motaln, Ana Rotter, Nadia A. Atai, Kristina Gruden, Cornelis J. F. Van Noorden, Tamara T. Lah

There are errors in S6 and S7 Files. The original files could not be opened. Please view the correct files below.

## Supporting Information

S6 File Selected differentially-expressed protease genes. (XLS)

S7 File Selected differentially-expresses protease inhibitor genes. (XLS)
