# Supplementary figures and images for: Expression Analysis of All Protease Genes Reveals Cathepsin K to Be Overexpressed in Glioblastoma
Source: PLoS One. 2014 Oct 30;9(10):e111819. doi: 10.1371/journal.pone.0111819 (PMC4214761; doi:10.1371/journal.pone.0111819)

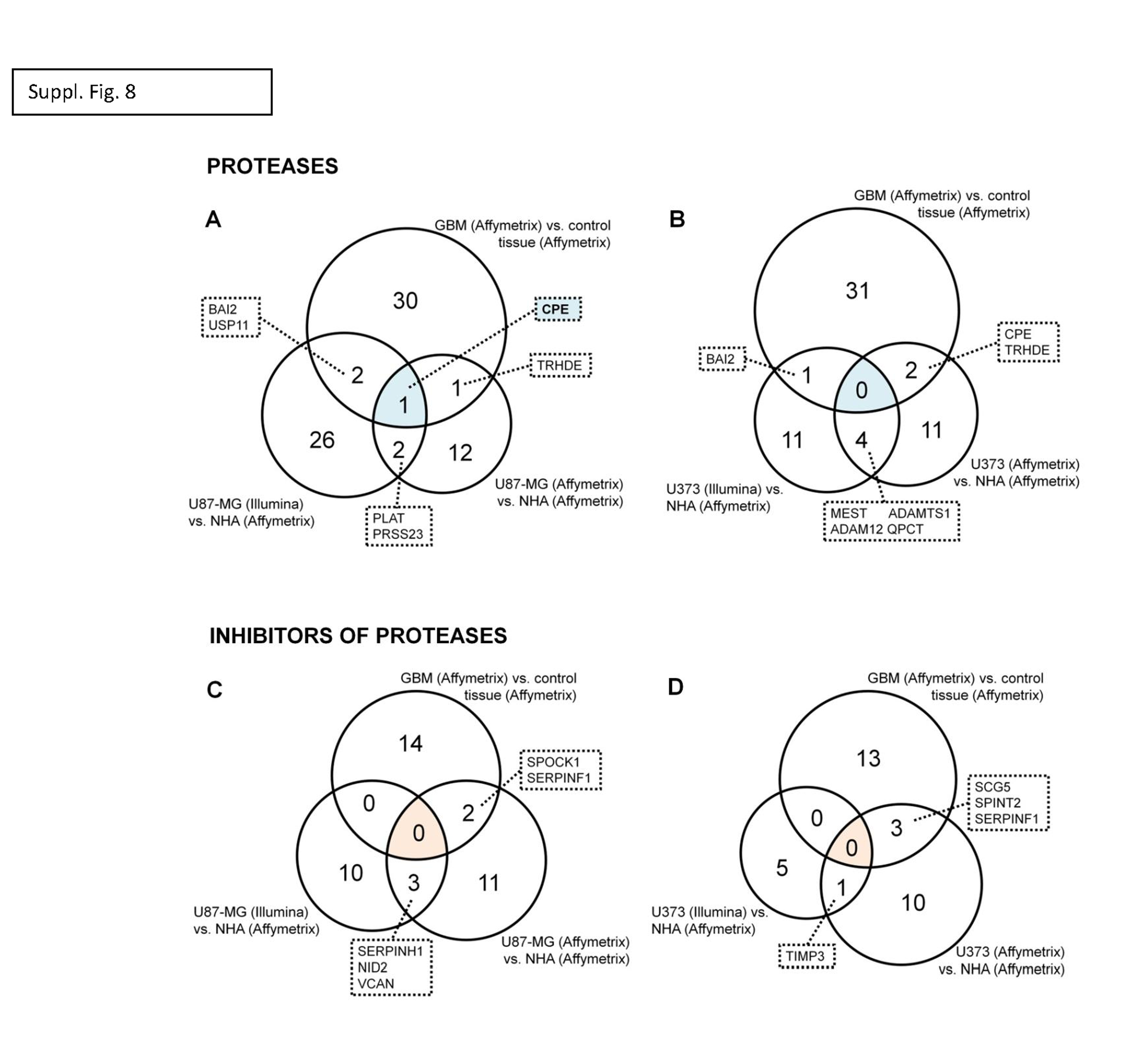

Supplement: Figure S1 — Venn diagrams of protease and protease inhibitor genes downregulated in both GBM tissue and GBM cells. In total, 669 protease and 242 protease inhibitor genes were checked for deregulation in GBM tissues and cells in comparison to non-malignant brain tissue and NHA cells. Venn diagrams include downregulated genes in GBM tissues and cells with PFP >0.05. Candidate genes appear in the intersections of Venn diagrams. Comparisons of protease genes downregulated in GBM tissue and in U87-MG cells (A) and downregulated in GBM tissue and in U373 cells (B) across Illumina and Affymetrx platforms and within the Affymetrix platform only are shown. The other 2 Venn diagrams show comparisons of protease inhibitor genes downregulated in GBM and in U87-MG cells (C) and downregulated in GBM and in U373 cells (D) across Illumina and Affymetrx platforms and within the Affymetrix platform only. Only one protease gene matched our selection criteria, CPE coding for carboxypeptidase E. (TIF) [file pone.0111819.s001.tif]

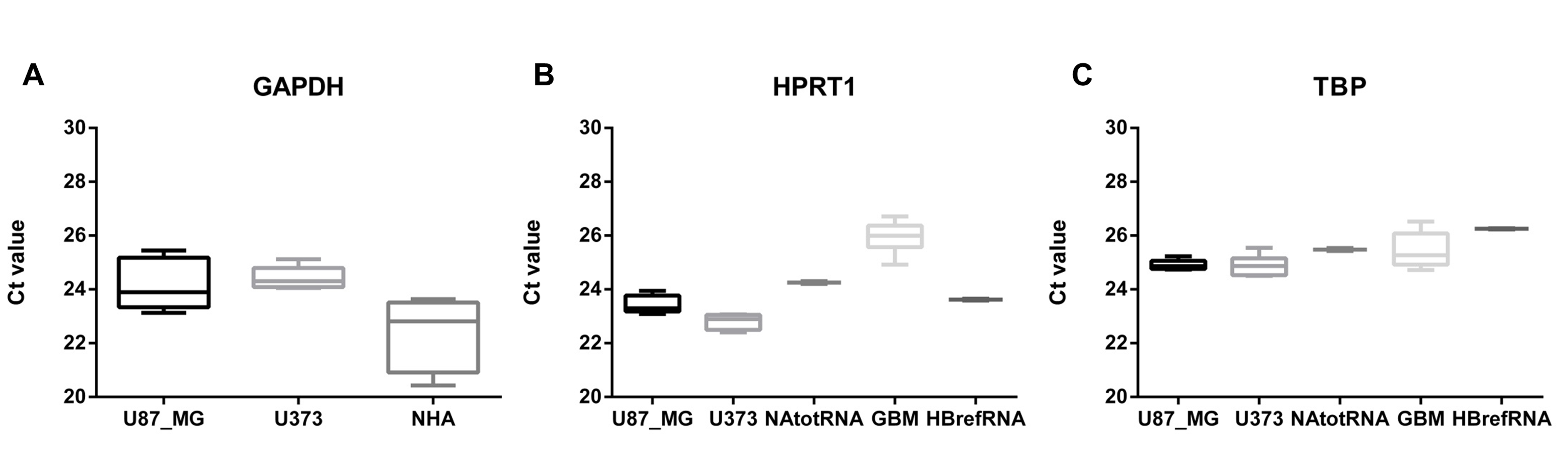

Supplement: Figure S2 — Boxplots of Ct values of reference genes (GAPDH, HPRT1 and TBP) used for RT-qPCR analysis. Median values are shown with box limits indicating the 25th and 75th percentiles as determined by R software; whiskers extend 1.5 times the interquartile range from the 25th and 75th percentiles and outliers are represented by dots. Sample points (biological replicates×technical replicates: n): A) nU87_MG = 6, nU373 = 6, nNHA = 6; B and C) nU87_MG = 6, nU373 = 6, nNAtotRNA = 2, nGBM = 16, nHBrefRNA = 2. (TIF) [file pone.0111819.s002.tif]

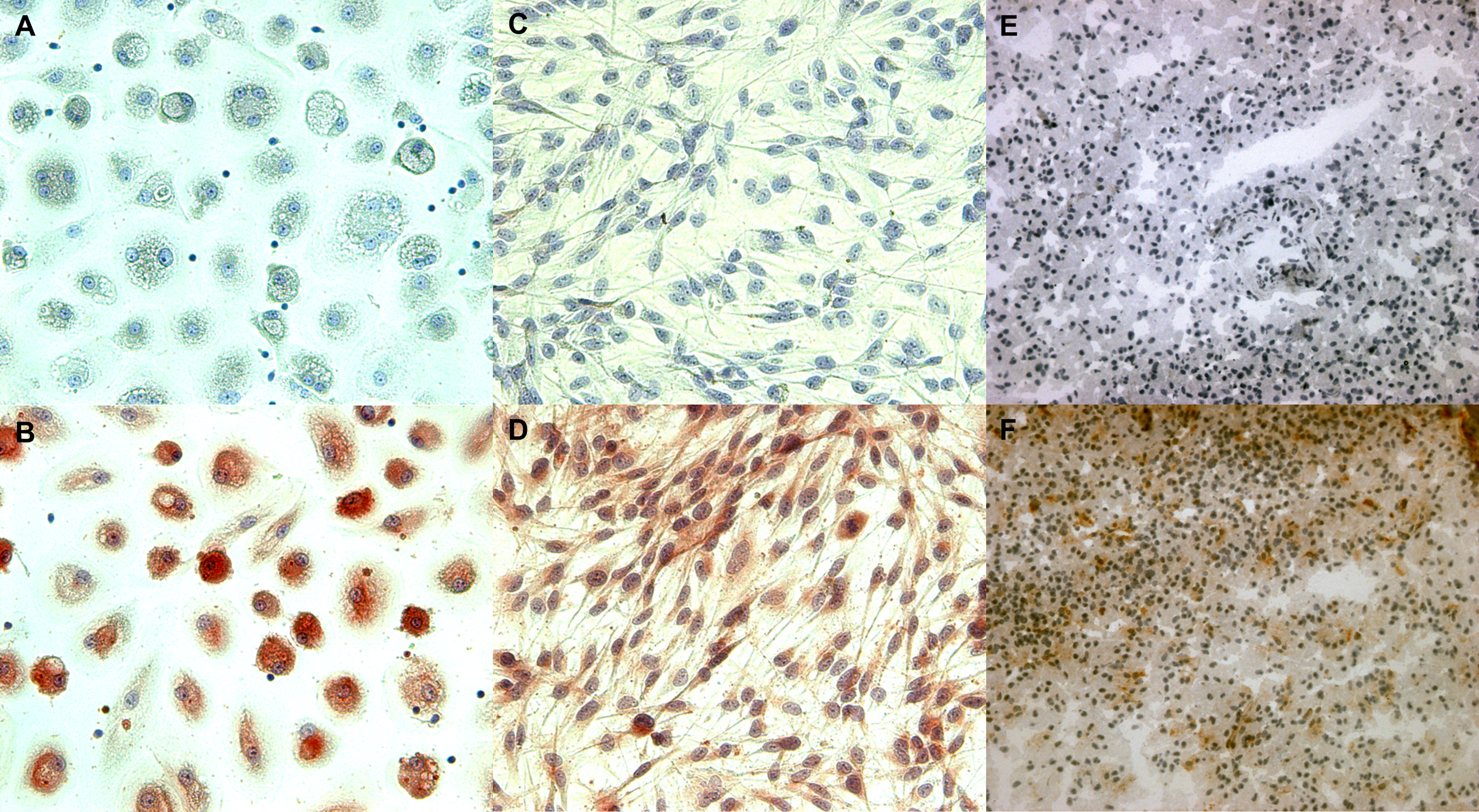

Supplement: Figure S3 — Immunohistochemical and immunocytochemical control staining. CatK staining was performed in the presence (B,D,F) or absence (A,C,E) of primary anti-CatK antibody. (A and B) osteoclasts in culture; (C and D) U373 cell line; (E and F) GBM tissue section. Magnifications: A–D, 200×; E–F, 100×. (TIF) [file pone.0111819.s003.tif]
